# Competitive Games

**Published:** 1871-11

**Authors:** 


### COMPETITIVE GAMES,

And all games which for victory depends on the rapidity or endurance of muscular action, are destructive to soul, body, and estate. The only kind of exercise which gives health and strength to the body, is that which is deliberate, slow, and continuous. It will benefit a man greatly to saw a cord of wood in a day, but to do it in an hour would be ruinous. The leisurely rowing of a boat for an hour or two is not objectionable, but to pull the oars with such violence as is common in all boat races is pernicious, dangerous, and even fatal.

Base-ball, cricket, and other forms of exercise which are used to "beat," are alike physically injurious, but

### A MORAL CURSE

attends the whole of them, because they are used in the form of competition, and thereby promote neglect of work, gossip, drinking, betting, and all the low forms of vice to which these things uniformly lead — to more or less of contentions, differences, altercations, profanities, fighting, and even murder itself.

The truth is, the spirit of gambling seems to be permeating all classes of society, which means the getting from your friend what belongs to him, without giving him any valuable thing in return; and when the stakes are large, there is an easy transition to the use of means which shall ensure successes founded in the deepest dishonor and in the foulest falsehoods. That is, the vices of Wall Street, which raise or depress the price of stocks, are spreading throughout our whole country, and are attaching themselves even to what were once joyous pastimes. Social and national safety, progress, and elevation, have their deepest and most secure foundations laid in the accumulation of money in legitimate business, in giving commodities for a fair valuation in money, where moderate profits are made on honest articles.

